# Quantitative trait locus mapping of genes associated with vacuolation in the adrenal X-zone of the DDD/Sgn inbred mouse

**DOI:** 10.1186/1471-2156-13-95

**Published:** 2012-11-06

**Authors:** Jun-ichi Suto

**Affiliations:** 1 Agrogenomics Research Center, National Institute of Agrobiological Sciences, Tsukuba, Ibaraki, 305-8634, Japan

**Keywords:** Adrenal X-zone, DDD mice, Quantitative trait locus (QTL) mapping

## Abstract

**Background:**

Adrenal gland of mice contains a transient zone between the adrenal cortex and the adrenal medulla: the X-zone. There are clear strain differences in terms of X-zone morphology. Nulliparous females of the inbred mouse DDD strain develop adrenal X-zones containing exclusively vacuolated cells, whereas females of the inbred mouse B6 strain develop X-zones containing only non-vacuolated cells. The X-zone vacuolation is a physiologic process associated with the X-zone degeneration and is tightly regulated by genetic factors. Identification of the genetic factors controlling such strain differences should help analyze the X-zone function. In this study, a quantitative trait locus (QTL) analysis for the extent of X-zone vacuolation was performed for two types of F_2_ female mice: F_2_*A*^*y*^ mice (F_2_ mice with the *A*^*y*^ allele) and F_2_ non-*A*^*y*^ mice (F_2_ mice without the *A*^*y*^ allele). These were produced by crossing B6 females and DDD.Cg-*A*^*y*^ males. DDD.Cg-*A*^*y*^ is a congenic mouse strain for the *A*^*y*^ allele at the agouti locus and is used for this study because a close association between the X-zone morphology and the agouti locus genotype has been suggested. The *A*^*y*^ allele is dominant and homozygous lethal; therefore, living *A*^*y*^ mice are invariably heterozygotes.

**Results:**

Single QTL scans identified significant QTLs on chromosomes 1, 2, 6, and X for F_2_ non-*A*^*y*^ mice, and on chromosomes 2, 6, and 12 for F_2_*A*^*y*^ mice. The QTL on chromosome 2 was considered to be because of the agouti locus, which has been suggested to be associated with X-zone vacuolation. A significant QTL that interacted with the agouti locus was identified on chromosome 8.

**Conclusions:**

The extent of X-zone vacuolation in DDD females was controlled by multiple genes with complex interactions. The murine X-zone is considered analogous structure to the human fetal zone. Therefore, the results of this study will aid in understanding function of not only of the X-zone but also of the human fetal zone. Identifying the genes responsible for the QTLs will be essential for understanding the molecular basis of X-zone function, which is currently unclear.

## Background

The mammalian adrenal cortex consists of three separate layers: zona glomerulosa, zona fasciculate, and zona reticularis. These layers have distinct endocrine roles. In rodents, an additional fourth, transient zone is present between the adrenal cortex and the adrenal medulla. In 1924, Masui and Tamura [1] first discovered a presence of a large transient zone at the innermost region of the adrenal cortex that could not be found anywhere else in the adult male cortex. This zone was later named the X-zone by Howard-Miller [2]. A close relationship was observed between X-zone morphology and gonadal functions. For example, the X-zone degenerates at puberty in males and at the first pregnancy in females.

There are apparent sex and strain differences in the morphology of the X-zone. With regard to sex difference, X-zone degeneration occurs earlier in males than in females; the X-zone disappears before 5 weeks of age in males, but gradually decreases in thickness with age in virgin females [3]. With regard to strain differences, inbred mouse strains can be classified into two categories based on the incidence and size of X-zones and the timing of X-zone vacuolation.

Nulliparous females of the inbred mouse DDD strain have thicker X-zones than the C57BL/6J females [3]. In DDD females, the X-zone degenerates with the accumulation of lipids; hereafter, this state is called a “vacuolated X-zone” after the designation given by Tanaka and Matsuzawa [3]. In contrast, in C57BL/6J females, the X-zone degenerates without the accumulation of lipids; hereafter, referred to as a “non-vacuolated X-zone” Strain differences are also observed with regard to the timing of X-zone degeneration. For example, fatty degeneration of the X-zone takes place earlier in A/Cam than in CBA/FaCam females [4].

Such clear sex and strain differences in the X-zone appearance suggest that the morphology of this zone is controlled both hormonally and genetically. Indeed, Shire and Spickett [4] reported that strain differences were a consequence of genetic variations at a single locus, “earlier X-zone degeneration locus” (*Ex*), on chromosome 7. Tanaka et al. [5,6] reported a close association between X-zone morphology and the agouti locus genotype on chromosome 2. They suggested that the *a* allele suppressed the vacuolation of the cells in the X-zone in a dominant manner. While such investigations have been useful, we propose that a genome-wide study would be a more beneficial approach.

Unfortunately, only one study has attempted a genome-wide search for genes controlling X-zone morphology. Di Curzio and Goldowitz [7] recently performed a QTL analysis on adrenal gland weight and structure using BXD recombinant inbred strains, and identified multiple significant and suggestive QTLs. In particular, they identified significant QTLs for X-zone width on chromosome 10 (*Mxzwdq1*) and 14 (*Mxzwdq2*) in males.

**Figure 1:**
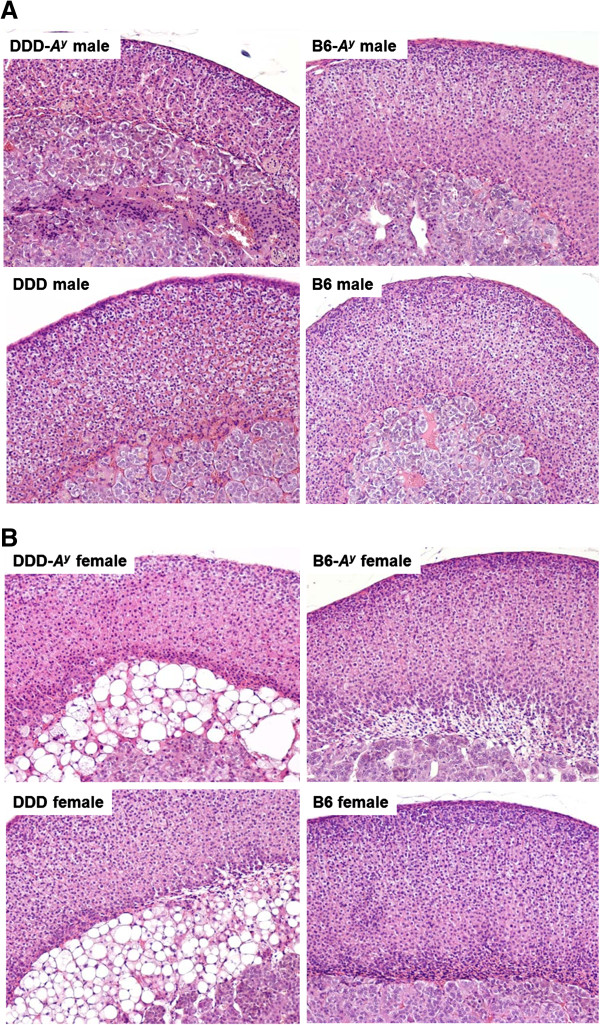
**Histological comparison of adrenal X**-**zone morphologies of DDD**-***A***^***y***^**, DDD**, **B6**-***A***^***y***^**, and B6 strains clearly indicating the differences between strains and between sexes.** (**A**) Male adrenal cortex: X-zone was absent in the four strains. Localized atrophy of the cortex or irregularly arranged cortex cells was observed in DDD-*A*^*y*^ and DDD males, but not in B6-*A*^*y*^ and B6 males. (**B**) Female adrenal cortex: X-zone was present in the four strains. Infiltration of fat cells with large fat droplets was observed in the X-zones of strains DDD-*A*^*y*^ and DDD, whereas no marked changes were observed in strains B6-*A*^*y*^ and B6.

The X-zones in DDD/Sgn females exclusively contain vacuolated cells, whereas those in C57BL/6J strain females contain only non-vacuolated cells (Figure 1). This difference may be controlled genetically. Identifying the genes responsible for the differences in the extent of X-zone vacuolation could provide clues for a better understanding of the critical role of this zone. The murine X-zone and the human fetal zone are considered analogous structures [8]. Therefore, the results of this study will aid in understanding function of not only of the X-zone but also of the human fetal zone. 

We have established a novel congenic strain for the *A*^*y*^ allele at the agouti locus (chromosome 2) in the inbred DDD/Sgn strain: DDD.Cg-*A*^*y*^. In normal mice, the agouti gene is expressed only in the skin [9,10], and it regulates pigmentation by serving as an inverse agonist of the melanocortin 1 receptor (MC1R) [11,12]. However, in *A*^*y*^ mice, the *A*^*y*^ allele is associated with a large deletion, causing agouti gene expression to be aberrantly controlled by the unrelated *Raly* gene promoter and leading to its ectopic overexpression [10,13-15]. As a result, *A*^*y*^ mice have a yellow coat. This makes it possible to distinguish the *A*^*y*^ mice from non-*A*^*y*^ mice visually. In addition, the *A*^*y*^ allele is homozygous lethal; therefore living *A*^*y*^ mice are invariably heterozygotes. DDD.Cg-*A*^*y*^ strain was used for this study because a close association between the X-zone morphology and the agouti locus genotype has been suggested [5,6]. While Tanaka et al. [5,6] investigated the effect of the *a*, *A*, and *A*^*w*^ alleles on the X-zone vacuolation, they did not investigate the effect of the *A*^*y*^ allele. Comparison of X-zone morphology between B6 and B6.Cg-*A*^*y*^, and DDD and DDD.Cg-*A*^*y*^ makes it possible to reveal the effect of the *A*^*y*^ allele. Furthermore, use of DDD.Cg-*A*^*y*^ strain together with C57BL/6J strain makes it possible to include the effect of the *A*^*y*^ allele in combination with the *a* and *A* alleles in the QTL analyses in F_2_ mice. The objectives of this study were to determine the genetic basis for strain differences in the extent of X-zone vacuolation between the DDD/Sgn and C57BL/6J strains and to clarify a possible effect of the agouti locus genotype on X-zone vacuolation.

## Methods

### Mice

The inbred mouse DDD/Sgn strain (hereafter designated DDD, coat color genotype is *AABBcc*) and the congenic mouse DDD.Cg-*A*^*y*^ strain (hereafter designated DDD-*A*^*y*^, *A*^*y*^*ABBCC*) were maintained at the National Institute of Agrobiological Sciences (NIAS). The inbred mouse C57BL/6J strain (hereafter designated B6, *aaBBCC*) was purchased from Clea Japan (Clea Japan, Inc., Tokyo, Japan). The congenic mouse B6.Cg-*A*^*y*^ strain (hereafter B6-*A*^*y*^, *A*^*y*^*aBBCC*) was purchased from The Jackson Laboratory (Bar Harbor, ME, USA). The DDD-*A*^*y*^ strain was established by introgressing the *A*^*y*^ allele from the B6-*A*^*y*^ strain in the DDD strain by backcrossing for 12 generations [16]. Hereafter, both DDD-*A*^*y*^ and B6-*A*^*y*^ are referred to as “*A*^*y*^ mice” Similarly, their control littermates, DDD and B6, are both referred to as “non-*A*^*y*^ mice” The *A*^*y*^ allele at the agouti locus (chromosome 2) is dominant and homozygous lethal; therefore, living *A*^*y*^ mice are invariably heterozygotes. *A*^*y*^ mice can be distinguished visually by their yellow coat color from non-*A*^*y*^ mice.

DDD-*A*^*y*^ males were crossed with B6 females to produce the F_1_ generation, and F_1_*A*^*y*^ (*A*^*y*^*aBBCC*) mice were intercrossed with F_1_ non-*A*^*y*^ (*AaBBCC*) mice to produce the F_2_ generation. F_2_ females were weaned at 4 weeks. The mice were housed in groups of 4–5 for 16 weeks.

All mice were maintained in a specific-pathogen-free facility with a regular light–dark cycle (12 h light and 12 h dark) and controlled temperature (23 ± 1°C) and humidity (50%). Food (CRF-1; Oriental Yeast Co., Ltd., Tokyo, Japan) and water were freely available throughout the experimental period. All animal experiments were performed in accordance with the guidelines of the Institutional Animal Care and Use Committee of NIAS.

### Phenotyping

At the age of 16 weeks, mice were euthanized with an overdose of ether and their adrenal glands were removed. Formalin-fixed tissues were embedded in paraffin, sectioned, and stained with hematoxylin and eosin.

**Figure 2:**
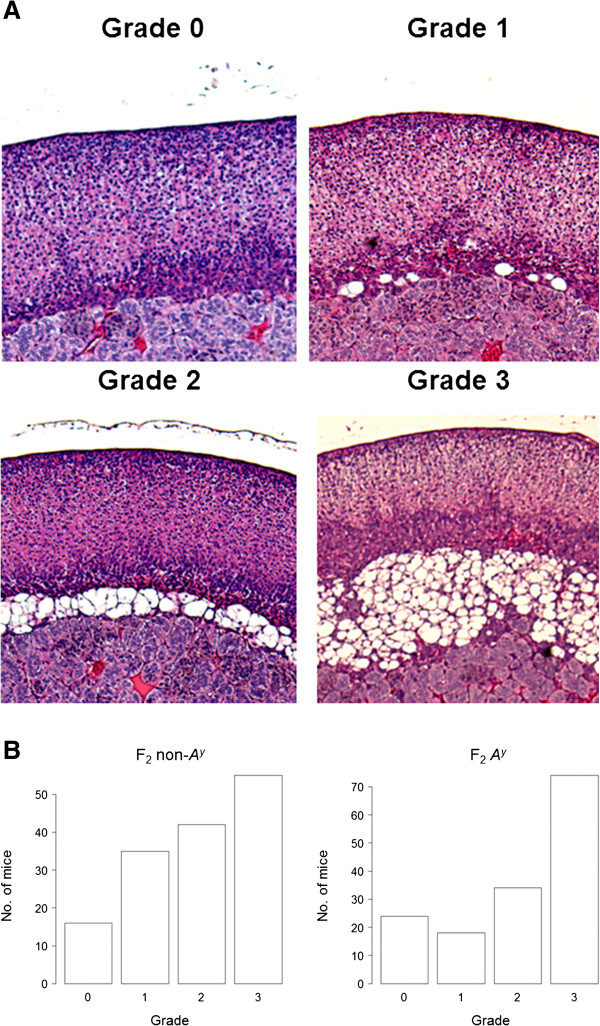
(**A**) **The extent of X-zone vacuolation was evaluated semi-quantitatively or qualitatively based on the following four-grade system.** “Grade 0” indicates that the X-zone contained only non-vacuolated cells; this corresponds to the X-zone morphology of the parental B6 (−*A*^*y*^) strain. The layer of vacuolated cells in the X-zone of “Grade 3” was thickest among the grades; this corresponds to the X-zone morphology in the parental DDD (−*A*^*y*^) strain. The layers of vacuolated cells in an X-zone of “Grade 1” and “Grade 2” were thinner than that of “Grade 3” The layer of vacuolated cells in an X-zone of “Grade 2” was apparently thicker than that in an X-zone of “Grade 1” (**B**) Histograms showing the grade distributions for F_2_ non-*A*^*y*^ and F_2_*A*^*y*^ mice. The mean ± S.E. grades for F_2_ non-*A*^*y*^ and F_2_*A*^*y*^ mice were 1.92 ± 0.09 and 2.05 ± 0.09, respectively. There was no significant difference between these two F_2_ types. The number of mice with grade 0 (absence of vacuolation) was 16 (10.8%) in F_2_ non-*A*^*y*^ and 24 (16.0%) in F_2_*A*^*y*^ mice.

The extent of X-zone vacuolation was evaluated semi-quantitatively or qualitatively. The width of the vacuolated layer of the X-zone could not be determined by a direct measurement because either the adrenal gland was not completely round, the X-zone was not always concentric, or a section was not necessarily made from the very center of the organ. According to Janat and Shire [17], CXB recombinant inbred strain mice can be divided into four types, including two parental types, based on X-zone morphology. Thus, the degree of X-zone vacuolation was evaluated quantitatively according to the following four-grade system (Figure 2A). 

“Grade 0” indicates that the X-zone contains only non-vacuolated cells; this corresponds to X-zone morphology in the parental B6 (−*A*^*y*^) strain. The layer of vacuolated cells in an X-zone of “Grade 3” is the thickest among the grades; this corresponds to X-zone morphology in the parental DDD (−*A*^*y*^) strain. The layer of vacuolated cells in an X-zone of “Grade 1” and “Grade 2” is thinner than that of “Grade 3” The layer of vacuolated cells in an X-zone of “Grade 2” is apparently thicker than that in an X-zone of “Grade 1”.

Alternatively, the occurrence of a vacuolated X-zone was evaluated qualitatively by regarding this as a binary trait. That is, an X-zone of “Grade 0” as indicated above was recognized as containing non-vacuolated cells only and X-zones of “Grades 1–3” were recognized as containing vacuolated cells.

In this study, the grade of X-zone vacuolation was treated in three different ways: as a parametric trait, as a non-parametric trait, and as a binary trait. QTLs that were identified as significant in at least one of these three analyses were considered to be significant.

### Genotyping and QTL analysis

Genomic DNA was isolated from the tails of mice using a commercial DNA extraction kit (Wizard Genomic DNA Purification Kit; Promega, Madison, WI, USA). Microsatellite sequence length polymorphisms were identified after PCR amplification of genomic DNA. PCR products were separated by 10% polyacrylamide gel electrophoresis and visualized with ethidium bromide straining.

Mice were genotyped for the following microsatellite marker loci: *D1Mit231*, *D1Mit303*, *D1Mit10*, *D1Mit102*, *D1Mit16*, *Apoa2* (see below), *D1Mit291*, *D2Mit312*, *D2Mit296*, *D2Mit92*, *D2Mit304*, *D2Mit285*, *D2Mit346*, *D3Mit203*, *D3Mit25*, *D3Mit212*, *D4Mit1*, *D4Mit178*, *D4Mit166*, *D4Mit234*, *D5Mit267*, *D5Mit113*, *D5Mit239*, *D5Mit161*, *D5Mit221*, *D6Mit116*, *D6Mit224*, *D6Mit188*, *D6Mit39*, *D6Mit108*, *D6Mit256*, *D6Mit259*, *D7Mit250*, *D7Mit362*, *D8Mit191*, *D8Mit205*, *D8Mit249*, *D8Mit183*, *D9Mit59*, *D9Mit191*, *D9Mit207*, *D9Mit198*, *D9Mit212*, *D10Mit188*, *D10Mit183*, *D10Mit42*, *D10Mit95*, *D11Mit236*, *D11Mit36*, *D11Mit124*, *D11Mit61*, *D12Mit136*, *D12Mit172*, *D12Mit156*, *D12Mit259*, *D12Mit141*, *D12Nds2*, *D13Mit207*, *D13Mit64*, *D13Mit110*, *D13Mit213*, *D13Mit171*, *D14Mit64*, *D14Mit193*, *D14Mit165*, *D15Mit174*, *D15Mit184*, *D15Mit193*, *D16Mit131*, *D16Mit57*, *D16Mit136*, *D16Mit139*, *D16Mit49*, *D17Mit164*, *D17Mit176*, *D17Mit139*, *D17Mit93*, *D17Mit123*, *D18Mit21*, *D18Mit149*, *D18Mit152*, *D18Mit25*, *D19Mit32*, *D19Mit91*, *D19Mit35*, *DXMit166*, *DXMit119*, *DXMit64*, and *DXMit38*. *Apoa2* (apolipoprotein A-II) was genotyped with a PCR-RFLP method according to the Suto et al. [18] procedure.

It should be noted that the chromosome 7 is divided into two parts. As a consequence of the introgression of the *Tyr* locus from the B6 strain, a mid-part of the DDD genome on chromosome 7 is replaced by a B6 genome in DDD-*A*^*y*^ mice. In this study, a region proximal to the B6 region was defined as “chromosome 7.1 (*D7Mit250*)” while a region distal to the B6 region was defined as “chromosome 7.2 (*D7Mit362*)”.

Of a total of 298 F_2_ females, 148 were F_2_ non-*A*^*y*^ and 150 were F_2_*A*^*y*^ mice. QTL analysis was done using R/qtl [19,20]. First, F_2_*A*^*y*^ and F_2_ non-*A*^*y*^ mice were analyzed independently. Threshold LOD scores for suggestive (P < 0.63) and significant (P < 0.05) linkages were determined by performing 1,000 permutations for each trait. For significant QTLs, a 95% confidence interval (CI) was defined by a decline of 1.5-LOD. After these single QTL scans, pair-wise evaluations for potential interactions between loci were made. At this stage, threshold LOD scores were based strictly on those recommended by Broman in “A brief tour of R/qtl” ( http://www.rqtl.org). Next, data for F_2_ non-*A*^*y*^ and F_2_*A*^*y*^ mice were combined and reanalyzed. The calculations for the threshold LOD scores were repeated. The presence or absence of possible statistical interactions between the genotypes at QTLs and the agouti locus (non-*A*^*y*^ and *A*^*y*^) was evaluated by two-way ANOVA.

The combined effects of all QTLs, including those that were significant and suggestive, were assessed using multiple QTL models. The percent of the variance explained by each QTL was reported based on these models.

### Statistics

Statistical analysis between two groups was performed using the Student’s or Welch’s t-test, or the Wilcoxon or Kruskal-Wallis test. Statistical analysis between more than two groups was performed using Tukey’s multiple comparisons tests with JMP8 software (SAS Institute Japan, Tokyo, Japan). P <0.05 was considered to be statistically significant.

## Results

### Comparisons of X-zone morphologies among the DDD-*A*^*y*^, DDD, B6-*A*^*y*^, and B6 strains

At the age of 16 weeks, X-zone morphologies in both sexes were compared among the DDD-*A*^*y*^, DDD, B6-*A*^*y*^, and B6 strains. Comparison of X-zone morphology between B6 and B6.Cg-*A*^*y*^, and DDD and DDD.Cg-*A*^*y*^ makes it possible to reveal the effect of the *A*^*y*^ allele. In agreement with previous findings, the X-zone was absent in males, irrespective of strain or agouti locus genotype (Figure 1A). Females had X-zones and as expected, there were gross strain differences in the extent of vacuolation. DDD-*A*^*y*^ and DDD females had X-zones that contained exclusively vacuolated cells, whereas B6-*A*^*y*^ and B6 females had X-zones containing only non-vacuolated cells (Figure 1B). X-zone vacuolation in all DDD-*A*^*y*^ (n = 4) and DDD (n = 2) females was scored as grade 3 (Figure 2A). In contrast, X-zone vacuolation in all B6-*A*^*y*^ (n = 4) and B6 (n = 2) females was scored as grade 0.

### X-zone vacuolation in F_1_ and F_2_ mice

X-zone vacuolation was also evaluated in (♀B6 × ♂DDD-*A*^*y*^) F_1_ and F_2_ mice. Because the agouti locus genotype of B6 strain was *a*/*a* and that of DDD-*A*^*y*^ strain was *A*^*y*^/*A*, the agouti locus genotype of F_1_ mice was either *A*/*a* (F_1_ non-*A*^*y*^ mice) or *A*^*y*^/*a* (F_1_*A*^*y*^ mice). In the similar way, F_2_ non-*A*^*y*^ mice had either *A*/*a* or *a*/*a* genotype and F_2_*A*^*y*^ mice had either *A*^*y*^/*a* or *A*^*y*^/*A* genotype (See also Materials and methods). All F_1_ non-*A*^*y*^ (n = 5) and all F_1_*A*^*y*^ (n = 5) mice had vacuolated X-zones of grade 1 or 2; the average grade was 1.6 in both F_1_ types.

Histograms showing these grade distributions in F_2_ non-*A*^*y*^ (n = 148) and F_2_*A*^*y*^ (n = 150) mice are shown in Figure 2B. The mean ± S.E. grades in F_2_ non-*A*^*y*^ and F_2_*A*^*y*^ mice were 1.92 ± 0.09 and 2.05 ± 0.09, respectively. There was no significant difference between these two F_2_ types. When the grade was treated as a binary trait, the number of mice with grade 0 (absence of vacuolation) was 16 (10.8%) in F_2_ non-*A*^*y*^ and 24 (16.0%) in F_2_*A*^*y*^ mice.

### QTL mapping

**Figure 3:**
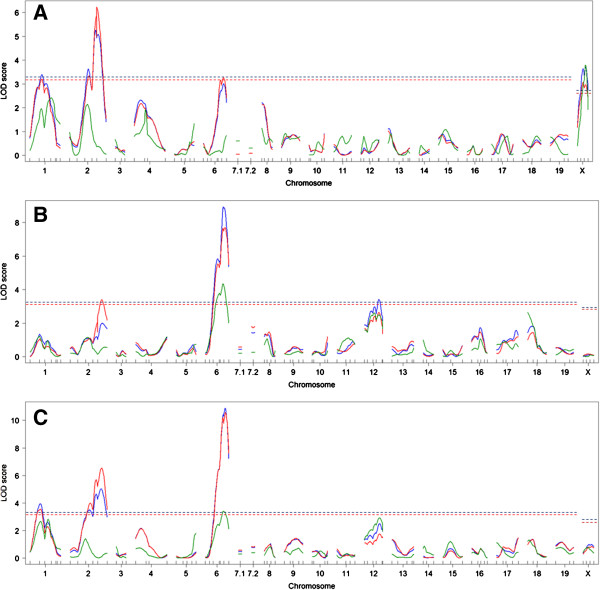
**Single QTL scan LOD score plots for X**-**zone vacuolation grades.** Results for (**A**) F_2_ non-*A*^*y*^ mice, (**B**) F_2_*A*^*y*^ mice, (**C**) combined F_2_ mice. The X-axis shows the chromosome numbers and the Y-axis shows the LOD scores at these locations. Blue lines indicate the LOD scores obtained when analyzing the grade as a parametric trait, red lines indicate the LOD scores obtained when analyzing the grade as a non-parametric trait, and green lines indicate the LOD scores obtained when analyzing the grade as a binary trait. Horizontal dashed lines (color-coded by each analytical method) indicate significant threshold LOD scores determined by 1,000 permutations.

**Figure 4:**
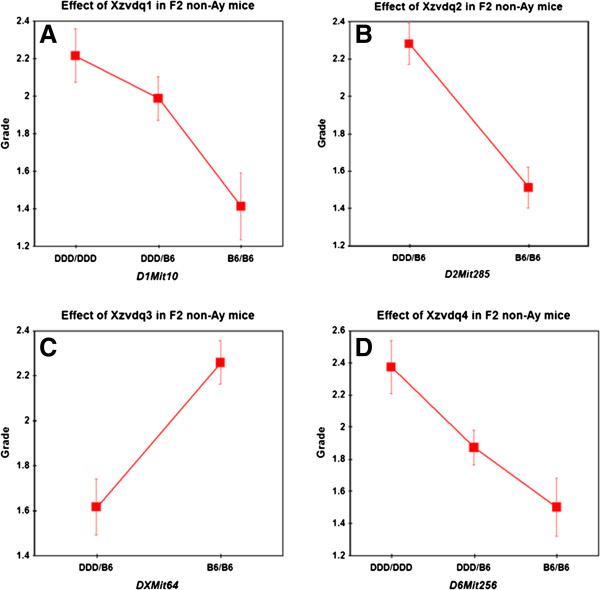
**Allelic contributions to QTLs for the X**-**zone vacuolation grades for F**_**2 **_**non**-***A***^***y ***^**mice (A–D).** Homozygous DDD alleles are represented by DDD/DDD, homozygous B6 alleles by B6/B6, and heterozygous alleles by DDD/B6. The Y-axes show the mean grade scores and the error bars are SE’s.

We first performed single QTL scans. When the grade was analyzed as a parametric trait in F_2_ non-*A*^*y*^ mice, three significant QTLs were identified on chromosomes 1, 2, and X. These loci were named *Xzvdq1* (X-zone vacuolation in DDD mice QTL no. 1), *Xzvdq2*, and *Xzvdq3*, respectively (Table 1 and Figure 3A). The DDD allele was associated with higher grades of vacuolation at *Xzvdq1* and *Xzvdq2*, whereas the DDD allele was associated with a lower grade of vacuolation at *Xzvdq3* (Figure 4A–C). When the grade was analyzed as a non-parametric trait, one significant QTL was newly identified on chromosome 6; this locus was named *Xzvdq4*. The DDD allele was associated with a higher grade of vacuolation at *Xzvdq4* (Figure 4D). When the grade was analyzed as a binary trait, *Xzvdq3* on X chromosome was the only significant QTL. 

**Table 1:** **QTLs identified for F**_**2 **_**non**-***A***^***y ***^**mice by single QTL scans**

**Method**	**Chromosome**	**Location (cM)**^**a**^	**95% CI (cM)**^**b**^	**Max LOD**^**c**^	**Nearest marker**	**High allele**^**d**^	**Name**^**e**^
parametric	1	40	17–69	3.40 *	*D1Mit10*	DDD	*Xzvdq1*
	2	73	65–90	5.26 *	*D2Mit285*	DDD	*Xzvdq2*
	4	24	7–72	2.32	*D4Mit178*	DDD	
	6	67	49–75	3.01	*D6Mit256*	DDD	
	8	21	21–41	2.22	*D8Mit191*	DDD	
	X	51	32–57	3.72 *	*DXMit64*	B6	*Xzvdq3*
non-parametric	1	39	14–68	3.20 *	*D1Mit303*	DDD	*Xzvdq1*
	2	76	68–87	6.24 *	*D2Mit285*	DDD	*Xzvdq2*
	4	25	7–75	2.17	*D4Mit178*	DDD	
	6	67	50–75	3.26 *	*D6Mit256*	DDD	*Xzvdq4*
	8	24	21–43	2.13	*D8Mit191*	DDD	
	X	43	30–57	3.07	*DXMit64*	B6	
binary	1	65	20–89	2.41	*D1Mit102*	DDD	
	2	50	2–69	2.14	*D2Mit92*	DDD	
	X	50	39–57	3.80 *	*DXMit64*	B6	*Xzvdq3*

a, Location denotes a chromosomal position showing a peak LOD score (in cM).

b, 95% CI is defined by a 1.5-LOD support interval.

c, Maximum LOD score for QTL. Significant LOD scores are indicated by *.

d, Allele associated with higher grade.

e, Assignment of QTL name is limited to significant QTLs.

**Figure 5:**
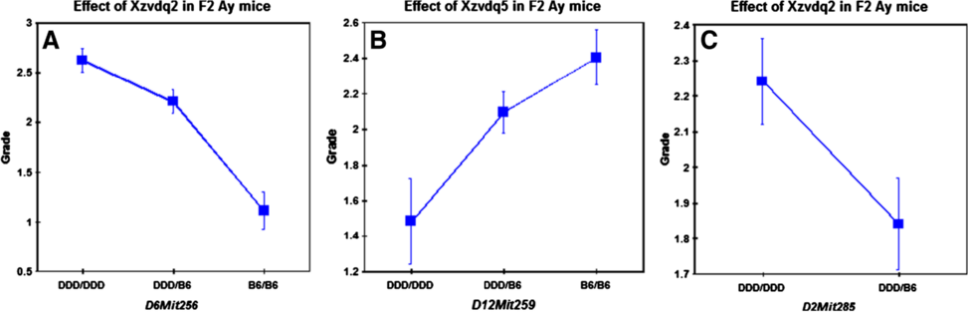
**Allelic contributions to QTLs for the X**-**zone vacuolation grades for F**_**2 **_***A***^***y ***^**mice (A–C).** Homozygous DDD alleles are represented by DDD/DDD, homozygous B6 alleles by B6/B6, and heterozygous alleles by DDD/B6. The Y-axes show the mean grade scores and the error bars are SE’s.

When the grade was analyzed as a parametric trait in F_2_*A*^*y*^ mice, two significant QTLs were identified on chromosomes 6 and 12. These loci were named *Xzvdq4* and *Xzvdq5*, respectively, because the former locus was located at a position similar to that identified in F_2_ non-*A*^*y*^ mice (Table 2 and Figure 3A, B). The DDD allele was associated with a higher grade of vacuolation at *Xzvdq4*, whereas the DDD allele was associated with a lower grade of vacuolation at *Xzvdq5* (Figure 5E, F). When the grade was analyzed as a non-parametric trait, one significant QTL was newly identified on chromosome 2. This locus was named *Xzvdq2* because this locus was located at a position similar to that identified in F_2_ non-*A*^*y*^ mice (Table 2 and Figure 3A, B). The DDD allele was associated with a higher grade of vacuolation at *Xzvdq2* (Figure 5G). *Xzvdq4* was the only significant QTL when the grade was analyzed as a binary trait. 

**Table 2:** **QTLs identified for F**_**2 **_***A***^***y ***^**mice by single QTL scans**

**Method**	**Chromosome**	**Location (cM)**^**a**^	**95% CI (cM)**^**b**^	**Max LOD**^**c**^	**Nearest marker**	**High allele**^**d**^	**Name**^**e**^
parametric	2	87	3–101	1.99	*D2Mit285*	DDD	
	6	60	55–70	8.95 *	*D6Mit256*	DDD	*Xzvdq4*
	12	52	22–62	3.42 *	*D12Mit259*	B6	*Xzvdq5*
non-parametric	2	87	72–101	3.40 *	*D2Mit285*	DDD	*Xzvdq2*
	6	65	53–73	7.71 *	*D6Mit256*	DDD	*Xzvdq4*
	12	52	13–62	2.63	*D12Mit259*	B6	
binary	6	59	37–71	4.36 *	*D6Mit256*	DDD	*Xzvdq4*
	12	39	13–62	2.60	*D12Mit156*	B6	
	18	9	9–32	2.64	*D18Mit21*	DDD	

a, Location denotes a chromosomal position showing a peak LOD score (in cM).

b, 95% CI is defined by a 1.5-LOD support interval.

c, Maximum LOD score for QTL. Significant LOD scores are indicated by *.

d, Allele associated with higher grade.

e, Assignment of QTL name is limited to significant QTLs.

Next, we combined the *A*^*y*^ and non-*A*^*y*^ progeny of the same F_2_ cross and performed single QTL scans. When the grade was analyzed as either a parametric or non-parametric trait, significant QTLs were identified on chromosomes 1, 2, and 6 (Table 3 and Figure 3C). *Xzvdq3* on chromosome X was no longer identified, even as a suggestive QTL. No significant QTLs were identified when the grade was analyzed as a binary trait. Additional analyses were done by using the agouti locus genotype as a covariate because the results of single QTL scans substantially differed between the two F_2_ types. Because the F_2_ non-*A*^*y*^ mice had *A*/*a* or *a*/*a* genotypes and the F_2_*A*^*y*^ mice had *A*^*y*^/*A* or *A*^*y*^/*a* genotypes at their agouti locus, these four genotypes were distinguished and included as covariates.

**Table 3:** **QTLs identified for combined F**_**2**_ (**F**_**2 **_**non**-***A***^***y***^**plus F**_**2**_***A***^***y***^) **mice by single QTL scans**

**Method**	**Chromosome**	**Location (cM)**^**a**^	**95% CI (cM)**^**b**^	**Max LOD**^**c**^	**Nearest marker**	**High allele**^**d**^	**Name**^**e**^
parametric	1	34	20–58	3.95 *	*D1Mit303*	DDD	*Xzvdq1*
	2	85	64–98	5.03 *	*D2Mit285*	DDD	*Xzvdq2*
	4	22	7–48	2.16	*D4Mit178*	DDD	
	6	65	56–72	10.90 *	*D6Mit256*	DDD	*Xzvdq4*
	12	54	13–62	2.50	*D12Mit141*	B6	
non-parametric	1	35	17–59	3.55 *	*D1Mit303*	DDD	*Xzvdq1*
	2	86	67–96	6.54 *	*D2Mit285*	DDD	*Xzvdq2*
	4	22	7–55	2.14	*D4Mit178*	DDD	
	6	66	55–73	10.58 *	*D6Mit256*	DDD	*Xzvdq4*
binary	1	55	18–71	2.82	*D1Mit102*	DDD	
	6	60	37–75	3.40	*D6Mit256*	DDD	
	12	54	13–62	2.91	*D12Mit141*	B6	

a, Location denotes a chromosomal position showing a peak LOD score (in cM).

b, 95% CI is defined by a 1.5-LOD support interval.

c, Maximum LOD score for QTL. Significant LOD scores are indicated by *.

d, Allele associated with higher grade.

e, Assignment of QTL name is limited to significant QTLs.

The agouti locus genotype was estimated by genotyping the genetic marker nearest to the agouti locus. The agouti locus is located at 76.83 cM (154.6 Mbp); therefore, *D2Mit285*, which is located at 75.41 cM (152.6 Mbp), was used for agouti locus genotyping. When the agouti locus genotype was included as an additive covariate, no additional QTLs were identified (Table 4). When the agouti locus genotype was included as an interactive covariate, one additional significant QTL was identified on chromosome 8; this locus was named *Xzvdq6*.

**Table 4:** **Summary of single QTL scans for combined F**_**2 **_**mice using the *agouti *genotypes as covariates**

**Chromosome**	**LOD scores[peak position (cM),name]**		
	***agouti*as an additive covariate (LOD**_**a**_**)**^**a**^	***agouti *as an interactive covariate (LOD**_**f**_**)**^**b**^	**LOD**_**i **_**(LOD**_**f**_-**LOD**_**a**_**)**^**c**^
1	4.03 (36, *Xzvdq1*)		
2	5.42 (80, *Xzvdq2*)	6.03 (82, *Xzvdq2*)	
6	11.22 (65, *Xzvdq4*)	11.89 (65, *Xzvdq4*)	
8		4.52 (24, *Xzvdq6*)	3.84 (23, *Xzvdq6*)

Only significant QTLs are listed.

a, Significant threshold LOD scores are 3.37 for autosomes and 2.76 for X chromosome.

b, Significant threshold LOD scores are 4.39 for autosomes and 3.43 for X chromosome.

c, LOD_i_ is the difference between the LOD score with *agouti* as an interactive covariate (LOD_f_) and the LOD score with *agouti* as an additive covariate (LOD_a_). It concerns the test of the QTL × *agouti* interaction. Significant threshold LOD scores are 2.41 for autosomes and 3.12 for X chromosome.

See also Figure 5.

**Figure 6:**
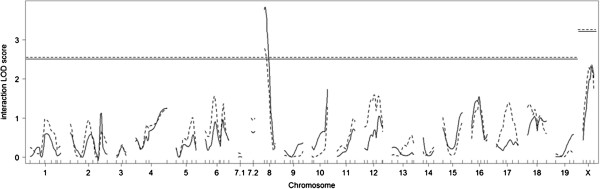
**Interaction LOD score plots for the X-zone vacuolation grades for combined F**_**2 **_**mice.** Horizontal dashed line indicates significant threshold LOD scores determined by 1,000 permutations. Solid lines denote the LOD scores when the four possible agouti genotypes (*A*/*a*, *a*/*a*, *A*^*y*^/*A*, and *A*^*y*^/*a*) were distinguished and included as covariates. Dotted lines denote the LOD scores when the two agouti genotypes (non-*A*^*y*^ or *A*^*y*^) were distinguished and included as covariates.

**Figure 7:**
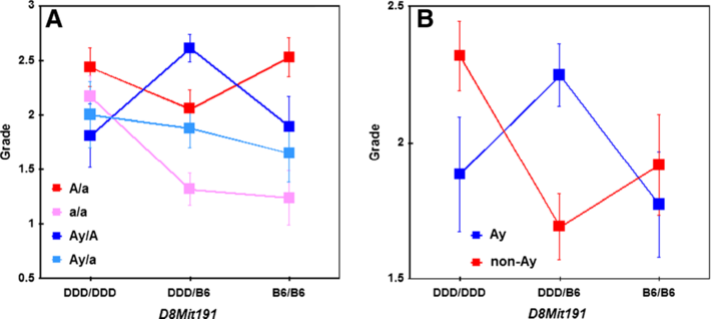
**Interaction effects between the agouti locus and *D8Mit191 *when the four possible agouti genotypes (*A*/*a*,*a*/*a***, ***A***^***y***^**/*A***, **and*A***^***y***^**/*a*) were distinguished (A)and when the two agouti genotypes (non-*A***^***y***^**or*A***^***y***^**) were distinguished (B).** Homozygous DDD alleles are represented by DDD/DDD, homozygous B6 alleles by B6/B6, and heterozygous alleles by DDD/B6. The Y-axes show mean grade scores and the error bars are SE’s. Y-axes represent the mean grade, and error bars represent SE.

Because the difference between the LOD score with the agouti locus genotype as an interactive covariate and the LOD score with the agouti locus genotype as an additive covariate concerns the test of the QTL × agouti locus genotype interaction, this test was done for combined F_2_ mice. One significant QTL was identified on chromosome 8 (Figure 6, LOD scores plotted as solid lines). The effects of interaction between agouti (*D2Mit285*) and *Xzvdq6* (*D8Mit191*) are shown in Figure 7A. Next, the agouti genotypes were classified into two categories: presence or absence of the *A*^*y*^ allele. Results of this analysis were very similar to those obtained above (Figure 6, LOD scores plotted as dashed lines). The effects of interaction between agouti (*D2Mit285*) and *Xzvdq6* (*D8Mit191*) are shown in Figure 7B. 

Next, possible interactions were analyzed pair-wise for F_2_ non-*A*^*y*^ and F_2_*A*^*y*^ mice. No significant pair-wise interactions were identified.

Finally, the relative contributions of each QTL when considered together were evaluated using multiple QTL models (Table 5). Most of the QTLs identified in the previous analyses were confirmed by this analysis. It should be noted that the LOD score for a locus on chromosome 8 in combined F_2_ mice increased to 4.85 when considering the simultaneous effects of other QTLs. The proportions of the total phenotypic variance accounted for in the F_2_ non-*A*^*y*^, F_2_*A*^*y*^, and combined F_2_ mice were 42.9%, 32.6%, and 34.8%, respectively.

**Table 5:** Multiple QTL analyses

**F**_**2 **_**type**	**Chr@cM**^**a**^	**df**^**b**^	**Type III SS**^**c**^	**LOD**	**%variance**^**d**^	**F value**	**P value**
non-*A*^*y*^	1@38.5	2	10.167	3.54	6.64	7.91	0.00056
	2@71.8	2	10.15	3.53	6.64	7.90	0.00057
	4@23.1	2	5.76	2.05	3.76	4.48	0.013
	6@65.5	2	10.42	3.62	6.81	8.11	0.00047
	8@21.2	2	3.65	1.32	2.39	2.84	0.062
	X@50.3	1	6.07	2.16	3.97	9.45	0.0026
*A*^*y*^	2@85.8	2	4.53	1.15	2.41	2.56	0.081
	6@59.5	2	37.34	8.43	19.91	21.13	9.13 × 10^−9^
	12@51.0	2	11.48	2.83	6.12	6.50	0.0020
combined	1@32.5	2	15.58	4.37	4.56	9.83	7.51 × 10^−5^
	2@83.8	6	28.61	7.82	8.37	6.01	6.22 × 10^−6^
	4@21.1	2	11.49	3.25	3.36	7.25	0.00085
	6@63.5	2	43.41	11.52	12.70	27.37	1.38 × 10^−11^
	8@23.2	6	17.58	4.91	5.14	3.69	0.0015
	12@53.0	2	6.23	1.79	1.82	3.93	0.021
	2@83.8:8@23.2	4	13.71	3.86	4.01	4.32	0.0021

a, cM position on the chromosome.

b, degrees of freedom.

c, sums of squares.

d, percentage of the total F_2_ phenotypic variance associated with each marker.

### The suggestive effect of the agouti locus genotype

As mentioned previously, a significant QTL, *Xzvdq2*, was identified on distal chromosome 2. There was a possibility that *Xzvdq2* was suggested to be due to agouti because some studies have suggested that the agouti locus was likely to have an effect on X-zone vacuolation [5,6]. In F_2_ non-*A*^*y*^ mice, those with the *A*/*a* genotype had a significantly higher grade of vacuolation compared with mice having *a*/*a* genotype based on the four-grade evaluation (Table 6). However, based on the all or none evaluation (i.e., binary 1 or 0), there was no significant difference between the two genotypes. In F_2_*A*^*y*^ mice, those with the *A*^*y*^/*A* genotype had a significantly higher grade of vacuolation than did mice with the *A*^*y*^/*a* genotype. Again, based on the all or none evaluation, no significant difference was identified between the two genotypes. 

**Table 6:** **Effect of agouti locus genotypes on the extent of X**-**zone vacuolation**

**F**_**2 **_**type**	**Trait**	**Mean grade** (**rank score**) **by agouti locus genotype**^**a**^		**Nominal P value**
		***A*/*a *(n=78)**	***a*/*a *(n=70)**	
F_2_ non-*A*^*y*^	parametric	2.28 ± 0.11	1.51 ± 0.11	2.33 × 10^−6^
	non-parametric	89.74	57.51	9.64 × 10^−7^
	binary	0.92 ± 0.03	0.86 ± 0.04	0.21
		***A***^***y***^***/A (n = 80)***	***A***^***y***^/***a (n = 70)***	
F_2 _*A*^*y*^	parametric	2.24 ± 0.12	1.84 ± 0.13	0.031
	non-parametric	83.51	66.34	0.00048
	binary	0.86 ± 0.04	0.81 ± 0.05	0.43

a, The agouti locus (76.83 cM, 154.6 Mbp) genotype was estimated by *D2Mit285* (75.41 cM, 152.6 cM) genotype. We also estimated the agouti locus genotype by *D2Mit494* (77.26 cM, 155.4 Mpb) genotype and performed statistical analyses, but the results did not differ greatly.

## Discussion

### Multigenic basis responsible for X-zone vacuolation

The extent of X-zone vacuolation in F_1_ mice was intermediate compared with the parental strains, and that in F_2_ mice was varied. Therefore, the extent of X-zone vacuolation is suggested to be a quantitative phenotype that is determined by multiple genes. Indeed, results of our QTL analyses revealed a multigenic basis for X-zone vacuolation.

Among the identified QTLs, *Xzvdq2* and *Xzvdq4* on chromosomes 2 and 6, respectively, were identified in both F_2_ types; the LOD scores for these QTLs increased in the combined F_2_ mice. Therefore, these QTLs were not disturbed by the *A*^*y*^ allele. In contrast, *Xzvdq1*, *Xzvdq3*, and *Xzvdq5* on chromosomes 1, X, and 12, respectively, showed high F_2_-type specificity (Tables 1, 2, and 3 and Figure 3A–C). In particular, *Xzvdq3* was not identified, even as a suggestive QTL, in combined F_2_ mice. Therefore, it was expected that the *Xzvdq3* would interact with the agouti genotype.

The interaction LOD score for *Xzvdq3* was certainly high, but was below the significant threshold (Figure 6). This suggests that the effect of *Xzvdq3* is in same direction in both F_2_ non-*A*^*y*^ and F_2_*A*^*y*^ mice, but is substantially weaker in the presence of the *A*^*y*^ allele. Intriguingly, the B6 allele at *Xzvdq3* on X chromosome was associated with a higher grade of vacuolation not only in the parametric analysis for F_2_ non-*A*^*y*^ mice, but also in the binary analysis. This indicates that the B6 allele exerts its effect more strongly than does the DDD allele for determining whether or not X-zone vacuolation develops.

Overall, four significant QTLs were identified for F_2_ non-*A*^*y*^ mice and three significant QTLs were identified for F_2_*A*^*y*^ mice. With regard to the number of QTLs responsible for X-zone vacuolation, Tanaka et al. [21] estimated that there would be three loci based on their study of SMXA recombinant inbred strains. The present results are in agreement with this estimation.

### The suggestive effect of the agouti locus genotype on the X-zone vacuolation

A putative genetic link between the agouti locus genotype and X-zone vacuolation was suggested by Tanaka et al. [5,6]. In the SM/J strain background at 70 days, mice with the *A*^*w*^/*A*^*w*^ genotype had X-zones containing highly vacuolated cells, whereas mice with the *A*^*w*^/*a* or *a*/*a* genotypes had X-zones containing only non-vacuolated cells [5]. These results suggested the *a* allele suppressed X-zone vacuolation in a dominant manner.

In the BALB/c strain background at 70 days, mice with the *A*/*A* genotype had X-zones containing highly vacuolated cells, whereas BALB/c strain mice with the *a*/*a* genotype (coisogenic mutant occurring in the BALB/c strain) had X-zones containing only non-vacuolated cells [6]. However, at 140 days, BALB/c mice with both the *A*/*A* and *a*/*a* genotypes had X-zones containing only vacuolated cells. These results suggested that the *a* allele was not only involved with the extent of X-zone vacuolation, but also with the timing of X-zone degeneration. It should be noted that B6 females never develop X-zone vacuolation regardless of age, which is in contrast to a BALB/c background [17].

In this study, the F_2_ non-*A*^*y*^ mice had *A*/*a* and *a*/*a* genotypes; the former was heterozygous and the latter was homozygous for the B6 allele. Similarly, the F_2_*A*^*y*^ mice had *A*^*y*^/*A* and *A*^*y*^/*a* genotypes; the former was homozygous and the latter was heterozygous for the DDD allele. When the vacuolation grade was analyzed as a parametric or non-parametric trait, significant differences were observed between the two genotypes in both F_2_ types (Table 6). The difference between genotypes was larger in F_2_ non-*A*^*y*^ mice than in F_2_*A*^*y*^ mice. In contrast to the Tanaka et al. proposal, the *a* allele suppressed vacuolation in a semidominant manner.

In this regard, it was also possible that the *A* allele promoted vacuolation. Thus, differences between the *A*^*y*^/*A* and *A*^*y*^/*a* alleles in F_2_*A*^*y*^ mice was not as strong as that between the *A*/*a* and *a*/*a* alleles in F_2_ non-*A*^*y*^ mice. In contrast, when the vacuolation grade was analyzed as a binary trait, the effect of the *a* allele was not significant in both F_2_ types. These results suggest that the agouti locus may have different genetic effects depending on different genetic background.

When the agouti locus genotypes were included as covariates by distinguishing four genotypes (*A*^*y*^/*A*, *A*^*y*^/*a*, *A*/*a*, and *a*/*a*), *Xzvdq6* was identified on chromosome 8 as an interaction locus. When the agouti locus genotypes were included as covariates by distinguishing two genotypes (*A*^*y*^ or non-*A*^*y*^), *Xzvdq6* was again identified. The patterns of LOD score plots were very similar for these two analyses. Therefore, whether or not mice had the *A*^*y*^ allele was critically important for the interaction with *Xzvdq6* on chromosome 8. This interaction may be one of the factors causing differences in the results in the aforementioned single QTL scans between F_2_ non-*A*^*y*^ and F_2_*A*^*y*^ mice.

These points of discussion are based on the assumption that *Xzvdq2* is due to the agouti locus. However, the possibility that *Xzvdq2* is due to another locus near to the agouti locus cannot be ruled out. Indeed, the agouti locus spans 259 kb on distal chromosome 2, and this region contains three other protein coding genes (*Raly*, *Eif2s2*, and *Gm14226*).

### Candidate genes or loci

Shire and Spickett [4] reported the presence of an “earlier X-zone degeneration locus” (*Ex*), on chromosome 7. The *Ex* locus is involved with the timing of X-zone degeneration rather than the extent of X-zone vacuolation; therefore, *Ex* is irrelevant to this study because the B6 strain never develops X-zone vacuolation regardless of mouse age [3,17,22]. The genomic region surrounding *Tyr* locus on chromosome 7 in DDD-*A*^*y*^ mice has been replaced by the B6 genome; DDD-*A*^*y*^ mice had X-zones containing vacuolated cells in a manner similar to DDD mice.

Janat and Shire [17] reported the presence of two gene loci that controlled X-zone development on chromosomes 12 (*Exz2*) and 16 (*Exz1*) in CXB recombinant inbred strains, which were derived from C57BL/6By and BALB/cBy strains. Inferring from their linkage analysis, *Exz2* may be located on distal chromosome 12; therefore, it may be allelic with the *Xzvdq5*.

Di Curzio and Goldowitz [7] addressed adrenal gland structure by QTL analysis. They analyzed X-zone widths in BXD recombinant inbred strains derived from the strains B6 and DBA/2J. They failed to identify any QTLs in females, but identified significant QTLs for X-zone width on chromosome 10 (*Mxzwdq1*) and 14 (*Mxzwdq2*) in males. Both of these QTLs did not coincide with those identified in this study.

Based on information retrieved from Mouse Genome Informatics, only a few genes or loci are currently recognized as having effects on the X-zone morphology. A possible candidate gene for *Xzvdq1* on chromosome 1 is KiSS-1 metastasis-suppressor (*Kiss1*) [23]. Nuclear receptor subfamily 0, group B, member 1 (*Nr0b1*, also known as *Dax1*) was also a possible candidate gene for *Xzvdq3* on the X chromosome [24]. In particular, *Nr0b1* was cloned as the gene responsible for X-linked congenital adrenal hypoplasia in humans [25,26]. Interestingly, loss of the function of *Nr0b1* gene in humans and mice results in the lack of degeneration of the fetal zone and X-zone, respectively [24-26]. For these reasons, the fetal zone and X-zone are considered analogous structures [8].

Because *Xzvdq6* interacts with an agouti genotype and is located on chromosome 8, the melanocortin 1 receptor (*Mc1r*) gene may be a possible candidate gene. *Mc1r* is located at the distal part of this chromosome (72.1 cM); therefore, *Xzvdq6* is unlikely to be allelic with *Mc1r* because 95% CI for *Xzvdq6* ranges from 21 to 34 cM. Adrenocortical dysplasia (*Acd*), which suppresses X-zone development, is also known to be located on chromosome 8. The *Acd* locus is at 53.04 cM on this chromosome and, therefore, is unlikely to be allelic with *Xzvdq6*.

X-zone morphology, including the extent of vacuolation, is determined genetically and, at the same time, modified hormonally [3]. Regarding hormonal factors, without doubt testosterone is critically important [27]. Testosterone administration induces X-zone degeneration, whereas gonadectomy preserves the X-zone in both sexes [28,29]. However, the X-zone degenerates in testicular feminized male (*Ar*^*Tfm*^/Y) mice, suggesting that androgen is not necessarily required for X-zone degeneration. Nevertheless, androgen is still a critical factor for X-zone degeneration; therefore, genes that are controlled by androgen are possible candidate genes for the identified QTLs.

## Conclusions

Despite efforts to elucidate critical roles of the X-zone, the physiological significance of this zone remains uncertain. Thus, it is most important to identify genes that account for the variations observed in X-zone morphology. Simultaneously, this study addressed the extent of X-zone vacuolation by adopting a genome-wide scan approach and showed that the extent of X-zone vacuolation in DDD females was controlled by multiple genes with complex interactions. The murine X-zone and the human fetal zone are considered analogous structures [8]. Therefore, the results of this study will aid in understanding function of not only of the X-zone but also of the human fetal zone. In addition, there is need to identify additional QTLs because there have been a limited number of genetic studies on adrenocortical morphology, including X-zone vacuolation. Identifying the genes responsible for the QTLs will be essential for understanding the molecular basis of X-zone function.

## Competing interests

The author declares that he has no competing interests.

## Authors’ contribution

JS has designed the research, carried out experiments for data collection, analyzed the data, and written the manuscript. The author read and approved the final manuscript.
